# *Rickettsia africae* and other unclassified *Rickettsia* species of the spotted fever group in ticks of the Western Ghats, India

**DOI:** 10.1007/s10493-023-00814-2

**Published:** 2023-06-22

**Authors:** N Naren Babu, Anup Jayaram, Amogh Milind Auti, Yuvraj Bhandari, Ujwal Shetty, Govindakarnavar Arunkumar

**Affiliations:** 1 Manipal Institute of Virology, Manipal Academy of Higher Education (Institution of Eminence Deemed to be University), Manipal, India; 2 Present Address: Department of Precision Medicine, University of Campania ‘Luigi vanvitelli’, Naples, Italy; 3 II-49, Vaikathu, Marotithota Road, Mooduathrady, Udupi District, Athrady, 576107 Karnataka India

**Keywords:** *Rickettsiae*, Ixodid ticks, Zoonosis, PCR, Western ghats, India

## Abstract

The spotted fever group (SFG) of *Rickettsia* are zoonotic disease-causing pathogens, commonly transmitted by hard ticks to a wide range of hosts, including humans. *Rickettsia conorii* is the common SFG recognised in India, whereas most of the infections due to other group species go undifferentiated at the species level. Hence, this study was conducted to screen host-seeking ticks in the Western Ghats region, India, for the DNA of SFG *Rickettsia.* The ticks were collected from Kerala, Goa, and Maharashtra states of India during a survey conducted between November 2017 and January 2018. In total, 288 tick pools were screened for *Rickettsia* spp. DNA using pan-*Rickettsia* real-time PCR, and conventional PCR targeting the *glt*A, *Omp*A and 17-kDa protein-coding genes. Nucleotide sequences were subjected to phylogenetic analysis using the NCBI BLAST tool to identify submitted sequences with higher homology. Neighbour-joining trees were constructed using the reference sequences of the GenBank database. Overall, *Rickettsia* spp. DNA was detected in 27.2% (62/228 pools) of host-seeking ticks across the Western Ghats region, with an estimated minimum infection rate of 0.057. Upon phylogenetic analysis, it was identified that the detected sequences were highly similar (> 99% sequence homology) to *R. africae*, *Candidatus* R. laoensis and an un-categorised *Rickettsia* species, and they were widely carried by *Haemaphysalis* ticks. The current study is the first report of *R. africae* and *Candidatus* R. laoensis in ticks in India. Although the pathogenicity of these species is not well documented, they may pose a potential threat to both animal and the human population in this geographical region.

**Supplementary Information:**

The online version contains supplementary material available at 10.1007/s10493-023-00814-2.

## Introduction

*Rickettsia* is an obligate intracellular Gram-negative bacterium that causes zoonotic diseases in a wide range of hosts (Raoult and Roux 1997; Weinert et al. 2009). *Rickettsia* species cause mild to severe infections in humans (Raoult and Roux 1997; Parola and Raoult 2001) – Rocky Mountain spotted fever (RMSF), Mediterranean spotted fever (MSF), scrub typhus, and epidemic typhus are a few of the widespread *Rickettsia* infections in humans (Raoult and Roux 1997; Parola and Raoult 2001; Parola et al. 2005, 2013). *Rickettsia prowazekii* and *R. rickettsii* are few among the highly pathogenic rickettsia species that may cause mortality (20–60%) among untreated human cases (Raoult and Roux 1997; Parola and Raoult 2001; Parola et al. 2013).

Many species of Rickettsiae are known for their endosymbiotic relationship with arthropods (Raoult and Roux 1997; Weinert et al. 2009). Among the arthropods carrying *Rickettsia*, ticks were strongly associated with the spotted fever group Rickettsiae (SFGR) (Rehácek 1989; Parola and Raoult 2001; Parola et al. 2005, 2013; Socolovschi et al. 2009). While several novel SFGRs are regularly being detected in ticks around the globe (Parola et al. 2013), many are known to cause rickettsiosis in humans (Raoult and Roux 1997; Parola et al. 2013). Some members of the SFGR, such as *R. africae* and *R. massiliae* are a few common tick-borne rickettsioses with high global prevalence. *Rickettsia africae* causes African tick-bite fever (ATBF) transmitted predominantly by *Amblyomma variegatum* among natives and travellers to Africa (Kelly et al. 1996; Raoult and Roux 1997; Jensenius et al. 2004; Parola et al. 2013; Binder and Gupta 2015). *Rickettsia massiliae* causes a Mediterranean spotted fever-like disease through *Rhipicephalus* spp. bite (Raoult and Roux 1997; Cascio et al. 2013; Parola et al. 2013). Both infections may present with fever, rashes, eschars and rarely with regional lymphadenopathy (Kelly et al. 1996; Cascio et al. 2013).

In India, *Rickettsia* infections have been recorded since World War II, and their reports have constantly increased in number over the last decade (Rathi and Rathi 2010; Dasari et al. 2014; Rahi et al. 2015). Infections of scrub typhus group (STG) and spotted fever group (SFG) *Rickettsia* are common across the country, especially in the sub-Himalayan region, Maharashtra, Rajasthan, Punjab and southern states of India (Mahajan et al. 2006; Batra 2007; Rathi and Rathi 2010; Dasari et al. 2014; Rahi et al. 2015). *Rickettsia conorii* transmitted by *Rhipicephalus sanguineus* sensu lato (s.l.) is the common SFGR in India, and it is prevalent in many states of the country (Rathi and Rathi 2010; Dasari et al. 2014), whereas records on other species are scanty. The current study assessed the presence and prevalence of SFGR carried by the host-seeking ixodid ticks of the Western Ghats region in India.

## Materials and methods

### Study area

#### Tick pools

The ticks collected from various parts of the Western Ghats (a highly diverged ecosystem of India) during a previous study (Naren Babu et al. 2019) were used for *Rickettsial* screening. In total, 8373 host-seeking ticks were collected (4474 larvae [53.4%], 3719 nymphs [44.4%], and 180 adults [2.2%]) through the flagging method from October 2017 to January 2018 (see details Naren Babu et al. 2019). Ticks of various genera, including *Haemaphysalis*, *Dermacentor*, *Amblyomma*, and *Rhipicephalus*, were identified morphologically. Ticks were collected from Sattari taluk, Goa (n = 1020), Dodamarg taluk, Maharashtra (n = 411) and Sultan-Bathery taluk, Kerala (n = 377). The ticks were pooled based on the site of collection, species and life stage, with a maximum of 15 ticks per pool. Pooled tick samples were homogenised using TissueLyser II (Qiagen, Valencia, CA, USA) and DNA was extracted using the QIAamp DNA Blood Mini Kit (Qiagen, Hilden, Germany) as per the manufacturer’s instructions.

#### Real-time PCR

The DNA extracted was screened for the presence of *Rickettsia* spp. using pan-*Rickettsia* real-time PCR. The primer, probe, reaction mix and cycling condition were optimised to amplify the 23 S rRNA region of *Rickettsia* as described by a previous study (Kato et al. 2013). The amplification was performed using an ABI 7500 real-time PCR machine (Applied Biosystems, Carlsbad, CA, USA). The reaction sets were validated using at least one internal positive control (IPC), and 2–3 non-template negative controls (using nuclease-free water). Cycle threshold values ≤ 40 were considered a positive cut-off for *Rickettsia*.

#### Conventional PCR

Further, the positive samples were amplified for *Omp*A, *glt*A and 17-kDa protein-coding genes as previously described (Anderson et al. 1988; Roux et al. 1997; Fournier et al. 1998; Stenos et al. 1998; Chmielewski et al. 2009). The reactions were carried out under the conditions described in Table 1, with standard enzyme activation (95 °C, for 7 min), extension (68 °C, 1 min) and final extension (72 °C, 7 min) using the ProFlex PCR system (Applied Biosystems). All the reaction cycles were conducted in a 25-µL reaction volume: which includes 10 µM of primer (1 µL each, forward and reverse), 10 µL buffer mix (Ambion Life Technologies, Carlsbad, CA, USA), 1.0 µL enzyme mix (Ambion Life Technologies) and 7.0 µL of nuclease-free water (NFW) and 5 µL of extracted query DNA. Each reaction set was validated using at least one synthetic positive control (referring *R. conorii* str. Malish 7), and 2–3 non-template negative controls (using nuclease-free water). The amplified products were resolved in 1.2% agarose gel, along with a molecular marker of 1 kb, to determine positive samples (Table 1).

**Table 1:** List of primers and cycling conditions used for the amplification of different *Rickettsia* spp. genes

Gene	Primer name	Primer sequence (5’→3’)	Denaturation (°C/s)	Annealing (°C/s)	Fragment length (bp)
*Omp*A (Fournier et al. 1998)	Rr. 190.70	ATGGCGAATATTTCTCCAAAA	95/20	56/30	630
	Rr. 190.701	GTTCCGTTAATGGCAGCATCT			
*glt*A (Roux et al. 1997)	RpCS.409d	CCTATGGCTATTATGCTTGC	95/30	45/30	750
	RpCS.1258n	ATTGCAAAAAGTACAGTGAACA			
17-kDa protein-coding gene (Anderson et al. 1988; Stenos et al. 1998)	Rr17.61p	GCTCTTGCAACTTCTATGTT	95/30	50/45	434
	Rr17.492n	CATTGTTCGTCAGGTTGGCG			
23 S rRNA Pan-Rickettsia real-time PCR (Kato et al. 2013)	PanR8_F	AGCTTGCTTTTGGATCATTTGG	95/15	60/60	221
	PanR8_R	TTCCTTGCCTTTTCATACATC TAG T			
	PanR8_P	Fl-CCTGCTTCTATTTGTCTT GCAGTAACACGCCA-BHQ1			

#### Sequencing

The PCR products were excised from the agarose gel post-electrophoresis and purified using the GenElute Gel Extraction Kit (Sigma-Aldrich, St. Louis, MO, USA). Purified products were sequenced with the same primer sets using Big Dye Terminator Cycle Sequencing Kit v.3.1 and 3500 Genetic Analyzer (both Applied Biosystems).

#### Phylogenetic analysis

The sequences were assembled from raw files using Sequencher v.5.4.6 (Gene Codes Corporation, Ann Arbor, MI, USA) and analysed through the NCBI BLAST tool (https://blast.ncbi.nlm.nih.gov/Blast.cgi). Further, phylogenetic trees were constructed with the query and *Rickettsia* reference sequences of *Omp*A and 17-kDa protein-coding genes using the Neighbour-joining method and Kimura-2 parameter at 1000 replicates of bootstrap. All the analyses were performed in MEGA v.11.

#### Statistical analysis

To understand the distribution of *Rickettsia* among the tick population, a minimum infection rate (MIR) and estimated pooled prevalence (EPP) were estimated for each tick species at the site level. The minimum infection rate was estimated as a ratio of the number of positive pools to the total number of ticks tested (Gu et al. 2003). Estimated pooled prevalence was estimated using a Bayesian approach and a Gibbs sampler iterative model by assuming pool size is 15 and an assay of perfect sensitivity and specificity (Cowling et al. 1999).

## Results

In total, 1808 ticks belonging to the genera *Haemaphysalis*, *Dermacentor*, *Amblyomma* and *Rhipicephalus* were grouped into 228 pools and screened for *Rickettsia* spp. DNA. Overall, 27.2% (62/228) of the pools were positive for pan-*Rickettsia* real-time PCR (Table 2). Among the *Rickettsia-*positive pools, 74.2% (46/62) were the ticks of immature life stages (i.e., 24 larval and 22 nymphal pools). The minimum infection rate (MIR) of *Rickettsia* in the tick pools was estimated at 0.057, with an adjusted prevalence rate of 0.022 (EPP). In general, *H. turturies* ticks had higher MIR in all the sites (Sattari, nymphs: 0.055; Dodamarg, adults: 0.333; Sultan-Bathery, adults: 0.285, nymphs: 0.375). However, the Bayesian adjusted prevalence estimation (EPP) revealed that *H. bispinosa* nymphs had higher *Rickettsia* spp. prevalence in Sultan-Bathery. Amongst the surveyed sites, Sultan-Bathery ticks showed as high as 0.09 MIR with 0.043 EPP (Table 2). In Sultan-Bathery, 66.7% of the larval population carried *Rickettsia* spp. DNA, and recorded the highest prevalence rate (0.13 EPP) amongst the tick population of all the sites.

**Table 2:** Site-wise positivity (%) of *Rickettsia* spp. among the tick population screened in the Western Ghats, India

Site of tick collection	Tick species and life-stage		Pools tested^1^	Positive pools^2^	Positivity (%)^3^	MIR^4^	EPP^5^ (mean ± SD)
Sattari taluk, Goa	All stages		98	21	21.42	0.02	0.017 ± 0.0036
	Adult	*H. spinigera*	5	-	-	-	-
		*H. turturies*	6	-	-	-	-
		*H. bispinosa*	5	-	-	-	-
		*H. minuta*	1	-	-	-	-
		*H. leachii*	2	-	-	-	-
		*Rhipicephalus* spp.	2	-	-	-	-
	Nymph	*H. spinigera*	24	6	25	0.02	0.022 ± 0.0085
		*H. turturies*	3	1	33	0.055	0.071 ± 0.15
		*H. bispinosa*	3	-	-	-	-
		*Amblyomma* spp.	1	-	-	-	-
	Larvae	*Haemaphysalis* spp.	42	14	33.33	0.023	0.029 ± 0.0076
		*Amblyomma* spp.	4	0	-	-	-
Dodamarg taluk, Maharashtra	All stages		54	6	11.11	0.014	0.0092 ± 0.0035
	Adult	*H. spinigera*	7	2	28.57	0.142	0.033 ± 0.02
		*H. turturies*	4	2	33	0.333	0.1 ± 0.18
		*H. bispinosa*	2	-	-	-	-
		*H. leachii*	1	-	-	-	-
		*Dermacentor* spp.	8	-	-	-	-
		*Rhipicephalus* spp.	2	-	-	-	-
		*Amblyomma* spp.	1	-	-	-	-
	Nymph	*H. spinigera*	12	-	-	-	-
		*H. intermedia*	2	-	-	-	-
	Larvae	*Haemaphysalis* spp.	14	2	14.28	0.006	0.015 ± 0.0091
		*Amblyomma* spp.	1	-	-	-	-
Sultan-Bathery taluk, Kerala	All stages		76	35	46.05	0.09	0.043 ± 0.0074
	Adult	*H. bispinosa*	21	10	50	0.344	0.048 ± 0.015
		*H. turturies*	5	2	40	0.285	0.051 ± 0.066
		*H. intermedia*	3	-	-	-	-
		*H. aculeata*	1	-	-	-	-
		*H. minuta*	1	-	-	-	-
		*H. centropi*	1	-	-	-	-
		*H. wellingtoni*	1	-	-	-	-
	Nymph	*H. spinigera*	5	-	-	-	-
		*H. turturies*	7	3	42.85	0.375	0.057 ± 0.13
		*H. bispinosa*	16	12	75	0.088	0.12 ± 0.11
		*H. intermedia*	2	-	-	-	-
		*H. cuspidata*	1	-	-	-	-
	Larvae	*Haemaphysalis* spp.	12	8	66.66	0.044	0.13 ± 0.2
Total	All stages		228	62	27.19	0.057	0.022 ± 0.028

^1^No. tick pools tested for pan-*Rickettsia* real-time PCR.

^2^No. tick pools producing cycle threshold (1) value of < 35 for pan-*Rickettsia* real-time PCR.

^3^Percentage of tick pools producing cycle threshold (1) value of < 35 for pan-*Rickettsia* real-time PCR of total tested

^4^Minimum infection rate (ratio of the number of positive pools to the total number of ticks tested)

^5^Estimated pooled prevalence using a Bayesian approach and a Gibbs sampler iterative model (assumed pool size is 15 and an assay of perfect sensitivity and specificity)

Out of the 62 *Rickettsia*-positive pools by real-time PCR, 13 were sequenced for *Omp*A, *glt*A and 17-kDa protein-coding genes, and the nucleotide sequences were submitted to GenBank under the following accession numbers: MK905239–MK905251 (*Omp*A), MN557213–MN557224 (*glt*A) and MN557225–MN557235 (17-kDa protein-coding gene). Further, the NCBI BLAST analysis identified the two query sequences extracted from *Haemaphysalis* spp. larvae of the population of Sattari taluk, Goa had homologous sequences to *R. africae* (> 99% identity). Two other detected *Rickettsia* sequences of Sultan-Bathery taluk, Kerala were homologous to the *Candidatus* R. laoensis (> 99% identity). The rest of the nine detected *Rickettsia* (four from Sattari, Goa, and five from Dodamarg, Maharashtra taluks) had homology with an unclassified *Rickettsia* species ZJ43/2007 (> 99% identity). Similar *Rickettsia* species have been reported earlier in the Kerala state of India in 2014 as an endosymbiont of *Rhipicephalus haemaphysaloides* (> 97% identity).

**Fig. 1:**
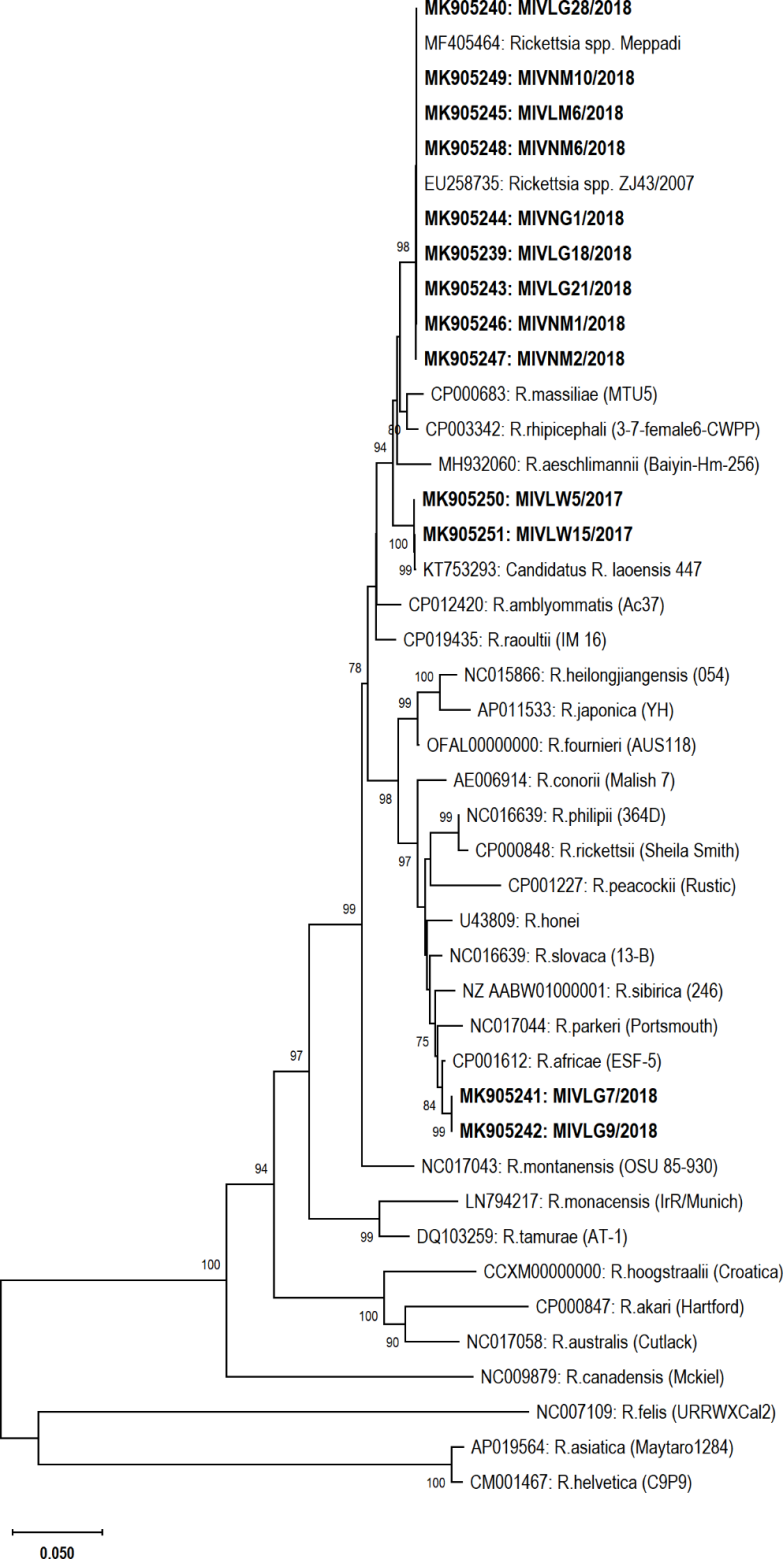
Phylogeny of *Rickettsia* species outer membrane protein-A gene sequences (519–624 bp) extracted from ticks of India (highlighted in bold) in comparison to the reference sequences. The phylogenetic tree was constructed using the neighbor-joining method based on the Kimura 2-parameter, analyzed at 1000 bootstraps (bootstrap values of > 70% are displayed at the nodes). The bar represents the divergence

**Fig. 2:**
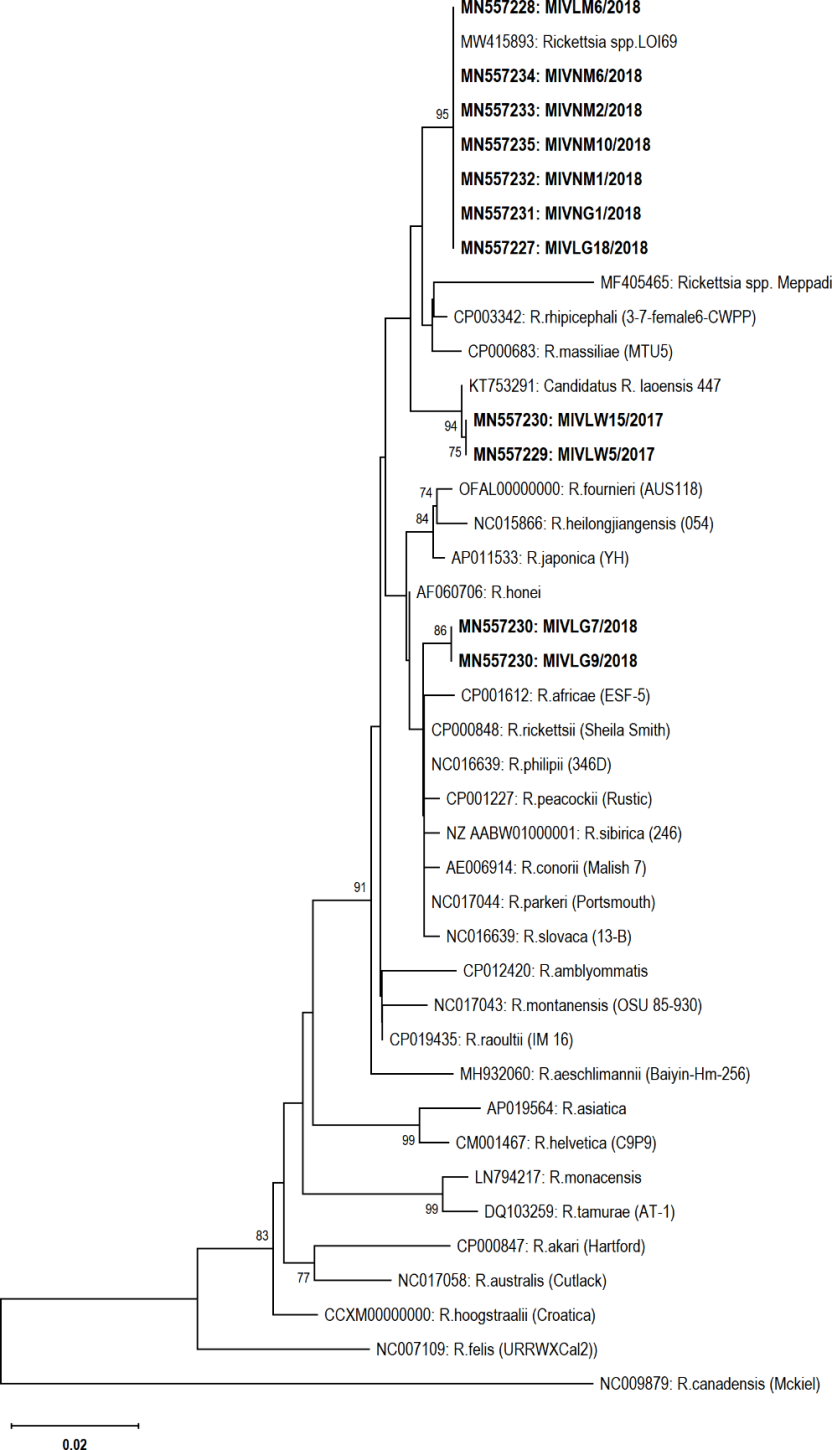
Phylogeny of *Rickettsia* species 17-kDa protein-coding gene sequences (432 bp) extracted from ticks of India (highlighted in bold) in comparison to the reference sequences. The phylogenetic tree was constructed using the neighbor-joining method based on the Kimura 2-parameter, analyzed at 1000 bootstraps (bootstrap values of > 70% are displayed at the nodes). The bar represents the divergence

Phylogenetic analysis showed that sequences in this study, LG07 and LG09 share the same clade with *R. africae*. LW05 and LW15 were found homologous with *Candidatus* R. laoensis (closely formed with *R. raoulti)* and seven other detected *Rickettsia* were found homologous with unclassified *Rickettsia* such as ZJ43/2007 and LOI69 (Figs. 1 and 2) (*Omp*A genes closely formed with *Rickettsia* endosymbiont of *R. haemaphysaloides* and *R. massiliae*).

Further, an additional phylogenetic tree was constructed using the *Omp*A genes of *R. africae* (Fig. S1). The tree formed two distinct clades; most of the sequences of clade-1 contain *R. africae* sequences extracted from *Amblyomma* ticks, whereas clade-2 contains sequences from diverse genera of vector species except *Amblyomma*. The query sequences were grouped in the clade-2, with close identity to an Egyptian strain.

## Discussion

The current study identified that SFGR is widely prevalent amongst the host-seeking ixodid ticks across the Western Ghats region in India. The ticks of the Sultan-Bathery site showed the highest MIR of 0.09, whereas the overall MIR was 0.057. Sultan-Bathery hosts half of the positive tick pools in this study. Higher *Rickettsia* positivity among larval tick pools of *Haemaphysalis* spp. is suggestive of the possible transovarial maintenance of these organisms in the tick population of the Western Ghats, India.

The current study provides the first evidence of the presence of *R. africae* in the larval stage of *Haemaphysalis* ticks, the widely prevalent cattle tick of the Western Ghats (Naren Babu et al. 2019). Sequence analysis revealed that the *Rickettsia* detected in this study is closest to the non-*Amblyomma* tick isolate of *R. africae*, and is closely related to the Egyptian strain isolated from *Hyalomma marginatum* (Fig. S1). In concurrence with other studies, *R. africae* is found to be prevalent among cattle ticks (Pillay et al. 2022) and occurs at a higher rate among larval tick population, inferring to possible transovarial maintenance of the species (Mazhetese et al. 2022). Only a few other reports from China had a similar prevalence of *R. africae* among the *Haemaphysalis* tick population (Fig. S1).

*Candidatus* R. laoensis (an *R. massiliae*-like species) is being reported in the larvae of *Haemaphysalis* spp. ticks of the Wayanad district for the first time in India. *Candidatus* R. laoensis was first and only described from the Nakai District of Laos in a *Haemaphysalis* nymph (Taylor et al. 2016). Additionally, an uncharacterised *Rickettsia* spp. (a novel *R. massiliae*-like species) was identified at multiple sites in the Western Ghats region. Similar species have been reported earlier from *Amblyomma testudinarium* in Laos (Satjanadumrong et al. 2019), R. *haemaphysaloides* in Taiwan (Satjanadumrong et al. 2019), and in India. Even though both the rickettsial species are widely prevalent across the ticks of the Western Ghats, further studies are required to determine their pathogenicity to mammals, humans and birds.

The diagnosis of acute febrile illnesses (AFI) has become increasingly challenging due to the constant spill-over of novel zoonotic pathogens into the human population (Parola and Raoult 2001; Weinert et al. 2009). In Asia, a vast proportion of AFI cases (8–80%) remain undiagnosed, leading to non-specific treatment (Susilawati and McBride 2014; Joshi and Kalantri 2015). Despite the SFGR infections frequently being detected in humans via the Weil-Felix test in India, the infecting species are mostly unknown (Rathi and Rathi 2010; Dasari et al. 2014; Rahi et al. 2015; Narvencar et al. 2017). Knowledge of the prevalence and distribution of *Rickettsial* pathogens is critical for early diagnoses, prompt treatment and disease control (Rathi and Rathi 2010; Dasari et al. 2014). The current report provides evidence for the prevalence of *R. africae*, *Candidatus* R. laoensis and another novel SFG *Rickettsia* among the tick population of India. This is suggestive of the potential risk of transmission of these SFGR to animals and/or humans through tick-bite. These SFGRs might also be the contributing cause for some proportion of pyrexia of unknown origin (PUO) in India. Therefore, accurate identification of the locally prevalent *Rickettsia* species in the tick population could help in improving the diagnosis of PUO.

## Electronic supplementary material

Below is the link to the electronic supplementary material.

Supplementary Material 1: **Figure S1**. Phylogeny of *Rickettsia* species *Omp*A gene sequences (519–624 bp) extracted from ticks of Goa, India (highlighted in bold) in comparison to various *Rickettsia africae* type strains submitted in GenBank. The phylogenetic tree was constructed using the neighbor-joining method based on the Tamura 3-parameter, analyzed at 1000 bootstraps (bootstrap values of > 60% are displayed at the nodes). The bar represents the divergence.

## Data Availability

The nucleotide sequences generated during the current study are available in the GenBank repository [GenBank ID for *Omp*A: MK905239 - MK905251, *glt*A: MN557213 -MN557224 and 17-kDa protein-coding gene: MN557225 - MN557235].
