# Supplementary material for: Rickettsia africae and other unclassified Rickettsia species of the spotted fever group in ticks of the Western Ghats, India
Source: Exp Appl Acarol. 2023 Jun 22;90(3-4):429–40. doi: 10.1007/s10493-023-00814-2 (PMC10406665; doi:10.1007/s10493-023-00814-2)
Supplement: Supplementary file 1 — Supplementary Material 1: Figure S1. Phylogeny of Rickettsia species OmpA gene sequences (519–624 bp) extracted from ticks of Goa, India (highlighted in bold) in comparison to various Rickettsia africae type strains submitted in GenBank. The phylogenetic tree was constructed using the neighbor-joining method based on the Tamura 3-parameter, analyzed at 1000 bootstraps (bootstrap values of > 60% are displayed at the nodes). The bar represents the divergence. [file 10493_2023_814_MOESM1_ESM.pdf]

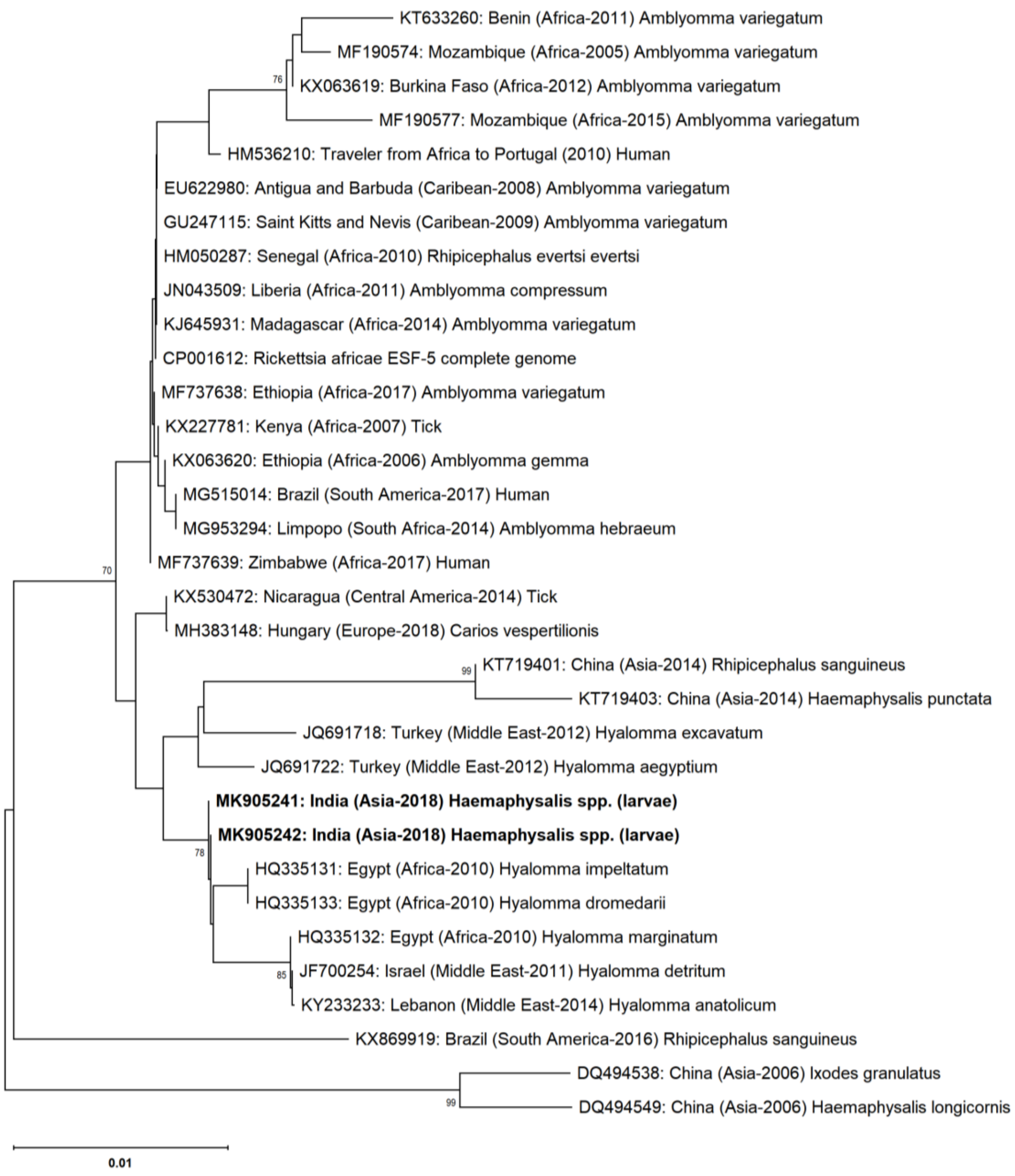


**Supplement figure 1. Phylogeny of *Rickettsia* species *Omp*A gene sequences (519-624 bp) extracted from ticks of Goa, India (highlighted in bold) in comparison to various *Rickettsia africae* type strains submitted in GenBank.**

*The Phylogenetic tree constructed using neighbor-joining method based on the Tamura 3- parameter, analyzed at 1000 bootstraps (bootstrap values of >60% were displayed at the nodes).* The bar represents the divergence.
